# Reducing risk for chronic disease: evaluation of a collective community approach to sustainable evidence-based health programming

**DOI:** 10.1186/s12889-024-17670-3

**Published:** 2024-01-20

**Authors:** Katherine Oestman, Ruth Rechis, Pamela A. Williams, Jill A. Brown, Katherine Treiman, Brittany Zulkiewicz, Michael T. Walsh, Karen Basen-Engquist, Trina Rodriguez, Catherine Chennisi, Amber Macneish, Alise Neff, Mike Pomeroy, Faiyaz A. Bhojani, Ernest Hawk

**Affiliations:** 1 The University of Texas MD Anderson Cancer Center, 7007 Bertner Ave Unit 1628, Houston, TX 77030 USA; 2 RTI International, 3040 East Cornwallis Road, P.O. Box 12194, Research Triangle Park, NC 27709-2194 USA; 3 City of Pasadena Texas Parks and Recreation Department, 3111 San Augustine Avenue, Pasadena, TX 77503 USA; 4 Harris County Public Health, 2223 West Loop South, Houston, TX 77027 USA; 5 Pasadena Independent School District, 1515 Cherrybrook Lane, Pasadena, TX 77502 USA; 6 Brighter Bites, 535 Portwall Street, Houston, TX 77029 USA; 7 Shell USA, Inc, 150 N. Dairy Ashford Road, Houston, TX 77002 USA

**Keywords:** Chronic disease, Population health, Implementation science, Risk reduction behavior, Program evaluation

## Abstract

**Background:**

Community initiatives can shape health behaviors, such as physical activity and dietary habits, across a population and help reduce the risk of developing chronic disease. To achieve this goal and impact health outcomes, Pasadena Vibrant Community aimed to engage communities in an ongoing dialogue about the importance of healthy behaviors, implement and advance community-based strategies to promote health, and improve diet and physical activity behaviors. The initiative was centered around a collaboration between a backbone organization, steering committee, and 7 collaborating organizations funded to implement multicomponent, evidence-based programs.. The common agenda was detailed in a community action plan, which included 19 interventions targeting healthy eating and active living among adults and youth in Pasadena, Texas.

**Methods:**

A mixed methods evaluation of the initiative was conducted over 4 years. Data sources included document reviews of quarterly progress reports (*n* = 86) and supplemental data reports (*n* = 16) provided by collaborating organizations, annual Steering Committee surveys (*n* = 4), and interviews conducted with staff from a subset of Collaborating Organizations (*n* = 4).

**Results:**

The initiative reached over 50,000 community members per year through 19 evidence-based interventions and impacted health outcomes, including knowledge and adoption of healthy eating practices and increased physical activity. Thirty-one systems-level changes were implemented during the initiative, including 16 environmental changes. Steering Committee meetings and shared goals enabled connections, communication, and cooperation, which allowed Collaborating Organizations to address challenges and combine resources to deliver their programs.

**Conclusions:**

Community initiatives can effectively permeate the community by reaching individuals, improving physical activity and dietary habits, and ensuring sustainability. Based on the experience reported here, the success of a community initiative can be facilitated if collaborating organizations come together to implement evidence-based interventions and tailor them to the community, and if they are empowered by significant leadership and supportive collaboration and aligned by a common agenda.

## Background

Heart disease and cancer have been two of the four leading causes of death in the US since 1900 [1]. Lifestyle factors, including diet and exercise, have been linked to chronic disease risk. Adherence to fiber and produce-rich diets such as the Mediterranean Diet have been associated with reduced risk of cardiometabolic disease including diabetes [2] and heart disease [3] and some cancers [4]. With regard to physical activity, meeting national recommendations of 150 min of moderate to vigorous physical activity (MVPA) per week has been linked to reduced risk of cardiovascular disease [5] and several cancers [6]. Physical activity and diet both influence the development of obesity, which has been linked to increased risk for several common cancers including breast, endometrial, colorectal and prostate cancer [7].

Increasing diet quality and MVPA are key targets for public health programs aiming to reduce cardiometabolic disease and cancer risk in communities. Several factors influence the diet and physical activity behaviors of populations, which can be mapped onto the social-ecological framework, which posits that there are factors influencing health across multiple levels, from individual and family factors to environment and policy [8]. Evidence-based interventions (EBIs) that encourage healthy eating and active living may offer effective prevention strategies when implemented at the population level, particularly when efforts span multiple levels of the social ecological model [9] and focus on communities experiencing health inequities.

Implementation and outcomes of EBIs in communities are influenced by local partnerships and infrastructure, program features, and contexts [10–12], emphasizing the importance of adapting programs and policies to local populations and settings [13]. EBI selection, adaptation, and implementation, may be successfully supported by multi-sectoral coalitions of community partners. Successful community coalitions for health are facilitated by strategies to build trust, incorporate and sustain the voice of the community [14, 15], and establish shared leadership and governance structures to build mutual accountability, collaboration, and implementation capacity [16]. The Collective Impact Framework offers guidance to develop community coalitions for health with the goal of creating longstanding commitments across diverse organizations from multiple sectors. The Collective Impact Framework centers around five key elements: common progress measures, common agenda, backbone organization, mutually reinforcing activities, and communication [17]. Together, the Collective Impact Framework informs the development of diverse community coalitions that have the capacity to select and implement EBIs with targets across the social-ecological framework.

The objective of this article is to describe a community intervention wherein healthy eating and active living EBIs were implemented through a multi-sector coalition — Pasadena Vibrant Community— and summarize program evaluation findings.

### Pasadena Vibrant Community

**Fig. 1:**
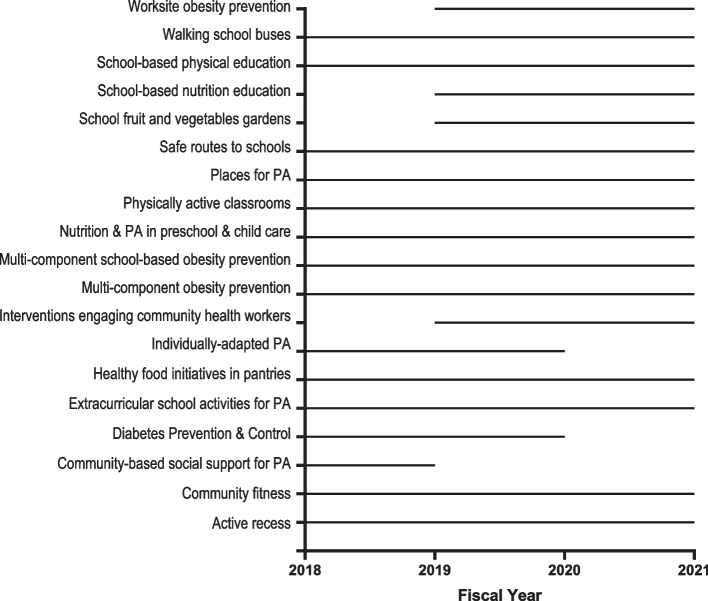
Pasadena Vibrant Community timeline

Pasadena Vibrant Community (PVC) is a whole-community cancer prevention program focused on promoting healthy eating and active living across multiple levels of the social ecological model. EBIs were selected, implemented, and evaluated by a multi-sector community coalition, or Steering Committee, guided by the Collective Impact Framework. PVC, implemented by The University of Texas MD Anderson Cancer Center’s Be Well Communities™ team, is an initiative of the Cancer Prevention and Control Platform. Be Well Communities is MD Anderson's place-based strategy for comprehensive cancer prevention and control working with communities to address modifiable risk factors for cancer. Described elsewhere [18], Be Well Communities utilizes a four-phase model (assessment, planning, implementation, sustainability) to build long-term partnerships with communities to deploy EBIs designed to meet the unique needs of target areas. Central to the model are creating strong community linkages, advancing professional and policy changes, establishing an active community coalition (e.g., a Steering Committee), and creating a sustainability plan to transition the initiative to the community. PVC took place from September 2014 through August 2021, and included the following steps: community assessment, program planning [including formation of the Steering Committee, development of a community action plan, and coordination of organizations (Collaborating Organizations) to implement EBIs], implementation of EBIs (September 2018- August 2021), evaluation activities, and sustainability planning (Fig. 1).

PVC applied the Be Well Communities model with residents of Pasadena, Texas, a majority-minority city of approximately 150,000 residents in the Greater Houston/Harris County area [19]. After initial community assessment, Pasadena was proposed as an implementation site for Be Well Communities based on both community need and capacity. Historically, residents of Pasadena have experienced high rates of chronic disease—for example, 30.3% experience high blood pressure and 12.0% of adults experience diabetes—coupled with high rates of sedentary behavior (30.5%) and obesity (32.5% of adults). Additionally, 38.7% of adults lack health insurance [20]. Pasadena also has many assets that support health, including parks, engaged residents, strong community-based organizations, and existing partnerships related to health-focused collaboration in the community for EBI implementation.

The PVC Steering Committee included representatives from school, local government, public health, healthcare, and social services sectors and was tasked with providing strategic guidance to the initiative and connecting the initiative to the community. The broad goals of PVC as determined by the Steering Committee were to (a) engage the community and initiate an ongoing dialogue about the importance of healthy behaviors, (b) create and advance community-based strategies to promote health, and (c) increase the adoption and maintenance of appropriate health behaviors related to diet and physical activity that can impact risk for chronic disease. The Be Well Communities team led the Steering Committee through a validation exercise of the preliminary community assessment and agreed on healthy eating and active living as intervention areas through a group consensus process. This process culminated in a comprehensive Community Action Plan (CAP), which served to define the common agenda of the Steering Committee. The CAP included a series of 19 EBIs supporting diet and physical activity based on evidence presented in the Robert Wood Johnson Foundation’s *What Works for Health Guide* [21] and the U.S. Department of Health and Human Service’s *The Community Guide* [22] (Table 1). PVC’s approach aimed to select healthy eating and active living EBIs that targeted multiple levels of the social ecological model (Table 1).

**Table 1:** Pasadena vibrant community’s evidence-based interventions for diet and physical activity, Pasadena, Texas, 2017 − 2021

Evidence-based Intervention	Collaborating Organization/*Sector(s) Involved*	Program and/or Activity
Active recess^a^	Pasadena Independent School District *School*	Recess packs, Playworks professional development, recess policy
Community fitness programs^a^	City of Pasadena Parks and Recreation Department *Local government, Recreation* YMCA of Greater Houston *Nonprofit*	Exercise for Life classes Walking clubs
Community-based social support for physical activity programs^a,b^	YMCA of Greater Houston *Nonprofit*	Walking clubs
Extracurricular activities for physical activity^a^	Pasadena Independent School District *School* YMCA of Greater Houston *Nonprofit*	Coordinated Approach to Child Health (CATCH) Kids Club Afterschool soccer clubs
Healthy food initiatives in food pantries^a^	Brighter Bites *Nonprofit*	The Brighter Bites program consists of three main components: (1) a weekly food distribution of 30 pounds of fresh produce per family; (2) nutrition education, in partnership with CATCH; and (3) a fun food experience during pickup time to involve parents and children.^c^
Diabetes Prevention and Control: Combined diet and physical activity promotion programs to prevent type 2 diabetes among people at increased risk^b^	YMCA of Greater Houston *Nonprofit* Memorial Hermann Community Benefit Corporation *Nonprofit*, *Medical*	Healthy Weight and Your Child^d^ Diabetes education classes
Interventions engaging community health workers^a,b^	Memorial Hermann Community Benefit Corporation *Nonprofit*, *Medical*	Community health worker
Individually adapted physical activity programs^a^	MD Anderson Center for Energy Balance *Medical*	Vibrant Lives program
Multicomponent obesity prevention interventions^a^	*Local government, Medical, Non-profit, School*	All EBIs in combination
Multicomponent school-based obesity prevention^a^	Pasadena Independent School District *School*	Modified CATCH program All other school-based interventions in combination
Nutrition and physical activity interventions in preschool and childcare^a^	Harris County Public Health *Local government, health*	OLE! (Outdoor Learning Environment)^e^ Texas Program
Physically active classrooms^a^	Pasadena Independent School District *School*	Physical Activity Specialist
Places for physical activity^a^	Pasadena Independent School District *School* City of Pasadena Parks and Recreation Department *Local government, Recreation*	Crosswalks at 6 elementary schools, Gardens Elementary Playground, Mae Smythe Elementary Playground, Young Elementary Playground, Sparks Elementary Track, Pasadena Healthy Parks Plan
Safe Routes to School^a,b^	Pasadena Independent School District *School* Harris County Public Health *Local government, health*	Safe Routes to School
School fruit and vegetable gardens^a,b^	Pasadena Independent School District *School*	School gardens
School-based nutrition education programs^a^	Pasadena Independent School District *School*	Modified CATCH program, integration of nutrition lessons into the core curriculum to align with the Texas Essential Knowledge and Skills (TEKS)
School-based physical education^a,b^	Pasadena Independent School District *School*	CATCH kids club (after school)^f^, kinesthetic learning, PE packs, PE curriculum
Walking school buses^a,b^	Pasadena Independent School District *School* Harris County Public Health *Local government, health*	Safe Routes to School
Worksite obesity prevention programs^a,b^	Pasadena Independent School District *School*	Worksite wellness

^a^Robert Wood Johnson Foundation. (2019). What Works for Health Guide. Retrieved July 19, 2021

^b^Community Preventive Services Task Force. The Guide to Community Preventive Services (The Community Guide). US Department of Health and Human Services. Accessed July 19, 2021

^c^Sharma SV, Markham C., Chow J., Ranjit N, Pomeroy M, Raber M. Evaluating a school-based fruit and vegetable co-op in low-income children: a quasi-experimental study. Prev Med. 2016;91:8–17

^d^YMCA of the USA. Healthy Weight and Your Child. 2022

^e^Texas Children in Nature Network. Outdoor Learning Environment (OLE! Texas)

^f^CATCH.org. CATCH Kids Club After School Curriculum Grades K-5. 2021

The Steering Committee identified seven community-based organizations – termed collaborating organizations—in Pasadena (detailed in Table 2) to receive funding to implement the EBIs from the CAP. These Collaborating Organizations, were selected because they addressed a unique need in the community, demonstrated the capacity to sustain work after the initiative’s funding ended, could conduct and report on program evaluation, had a track record of delivering and evaluating EBIs, and were Steering Committee members. Within the CAP, each Collaborating Organization had specific annual goals related to implementation and evaluation of the EBIs. Consolidated across years and organizations, EBIs aimed to increase access to physical activity among local school students and community residents, increase nutrition education in local schools, increase fruit and vegetable intake among local school students, and achieve clinically significant weight loss among overweight and obese school employees.

**Table 2:** Pasadena Vibrant Community’s Collaborating Organizations, Pasadena, Texas, 2017 − 2021

Collaborating Organization	Description
Brighter Bites	Brighter Bites is a Houston-based nonprofit that delivers a comprehensive, multicomponent school program that increases access to fresh vegetables and fruits, combined with nutrition education, for obesity prevention among children from low-income homes and their families. Their goal is to help curb the childhood obesity epidemic in Texas by increasing demand for fresh vegetables and fruits, leading to better family eating habits and improved health outcomes
City of Pasadena Parks and Recreation Department (PARD)	PARD has served the citizens of Pasadena since its establishment in 1964. PARD has been and is committed to maintaining a safe environment for all while providing programming, facilities, and relationships that enrich and enlighten the lives of all families in Pasadena. PARD oversees 42 parks and 4 recreation centers
Harris County Public Health (HCPH)	HCPH is the health department for the unincorporated area of Harris County, Texas, and its 33 independent municipalities: a jurisdiction of 2.1 million people, which includes Pasadena. Guided by the principles of innovation, engagement, and equity, HCPH was named Local Health Department of the Year in 2016. HCPH is committed to deploying resources that support people to be healthy. In addition to providing public health services, HCPH also manages community health improvement efforts, including the Healthy Living Matters (HLM) initiative to curb childhood obesity. HLM Pasadena was a coalition of Pasadena area stakeholders and community members with an interest in the health and wellness of the community who convene to secure funding, add additional support, and build capacity for Pasadena
MD Anderson Center for Energy Balance	The Center for Energy Balance in Cancer Prevention and Survivorship was founded to help bridge the gaps in knowledge surrounding the relationships between physical activity, nutrition, obesity, and cancer. The Center uses advancements in the energy balance research field to improve interventions that modify unhealthy behaviors in people at risk for cancer, patients, and survivors, and to deliver innovative change at the clinical level
Memorial Hermann Community Benefit Corporation (MHCBC)	MHCBC leads population health efforts for the Memorial Hermann Health System. They implement initiatives built on the foundation of 4 intersecting pillars—access to healthcare, emotional well-being, food as health, and exercise is medicine—taking them outside their campuses and into the community
Pasadena Independent School District (PISD)	PISD is among the 30 largest school districts in Texas and one of the fastest growing. It is a majority-minority school district, with 89% of the 55,397 students from a minority group and 77% of students are economically disadvantaged, 60% are considered academically at-risk, and close to 30% are English Language Learners. The mission of PISD is to create unlimited opportunities for students by empowering them to be the best they can be. The district was an early adopter of coordinated school health programming, making investments in teacher training and hiring staff at the district level to address the complex needs of the whole child
YMCA of Greater Houston	As a well-established organization across the US, the YMCA has an incredible track record for delivering and evaluating their evidence-based programs. Sports teach teamwork, discipline, and how to win graciously and how to lose honorably. These are the types of lessons that benefit young people for the rest of their lives. Organized youth sports, such as soccer, are also positively associated with strong academic performance, low juvenile arrest rates, and low teen birth rates. Beyond the social benefits, research indicates that access to publicly provided recreation programs can reduce children’s risk of becoming overweight and obese

PVC prioritized public schools as a key Collaborating Organization. Schools have long-offered a foundational venue for obesity prevention efforts [23, 24]. However, schools also offer reach into the community through employees and parents, and school-based healthy eating and active living programs benefit from engaging other community assets to expand and reinforce health promotion opportunities and messaging [25]. The CAP was structured around the catchment area of Pasadena Independent School District (PISD), and the school district served as a central hub for PVC. PISD shared broad communications across the district, engaged community members in the School Health Advisory Committee, and hosted events to encourage the entire district to get active and healthy.

Additionally, PISD and other Collaborating Organizations supported each other’s efforts (e.g., hosting walk-to-school days, serving on the Pasadena Healthy Parks Plan committee), emphasizing the tenets of Collective Impact to work collaboratively around a common agenda (the CAP detailing 19 EBIs as shown in Table 1) by using reinforcing activities and measurement systems supported by a backbone organization [17]. The Be Well Communities team at MD Anderson served as the backbone organization [17] that led the Steering Committee; aligned Collaborating Organizations’ activities and programs to implement the CAP; established working groups to support day-to-day implementation of programs and enhance communication between partners; and evaluated the initiative in collaboration with its contracted evaluation partner, RTI International.

## Methods

RTI International and the Be Well Communities team collaboratively developed the evaluation plan and used a mixed methods approach, collecting data from multiple sources to explore and assess partnerships and implementation processes and outcomes. A mixed methods approach incorporates both quantitative and qualitative data to provide a more comprehensive understanding than can be achieved through either method alone [26]. Community programming is under the purview of the institutional Quality Improvement Assessment Board (QIAB) and this initiative was approved by the MD Anderson QIAB. Informed consent was obtained by Collaborating Organizations from program participants.

The evaluation sought to answer the following questions:

1. What was the reach of the PVC programs, defined as proportion of total community exposed to one or more EBIs?h
2. How have the PVC programs affected individual level changes- healthy eating and physical activity (PA) related knowledge, fruit and vegetable consumption, MVPA, and weight management metrics among participants?
3. How has PVC affected systems-level changes—such as professional practice, funding, policy, and infrastructure/environment?
4. What organizational partnerships have developed because of PVC?
5. What is the collective impact of PVC?

The evaluation synthesized quantitative and qualitative findings from quarterly reports provided by Collaborating Organizations (including self-report assessments of EBI participant PA and diet knowledge and PA and diet-related behaviors as well as self-reported anthropometrics), an annual survey of Steering Committee members, stakeholder interviews with Collaborating Organizations, all of which are described below.

### Reach

#### Collaborating organization reports

Collaborating Organizations submitted quarterly reports to document aggregate-level data on program implementation progress, reach, and impact on health-related outcomes and implementation of systems-level changes (e.g., professional practice, funding, policy, and environment). Supplemental reports were gathered when data reporting was more in-depth than the reporting template allowed. RTI conducted an annual document review of all reports. Over four years of the initiative, RTI reviewed 86 quarterly reports and 16 supplemental reports from Collaborating Organizations. This data synthesis focused on tracking progress of Collaborating Organizations’ planned activities and answering evaluation questions 1, 2, and 3. Reach was calculated as the extent to which each intervention served its intended audience [27, 28]; specifically, the number of participants directly impacted by each intervention in the CAP divided by the total number of Pasadena residents (I.E., 153,286).

Systems-level changes related to Collaborating Organization professional practice, funding, policy, and infrastructure/environmental changes were also gleaned from the quarterly reports. Professional practice changes included adjustments to workflows or staff trainings. Funding changes were related to adjustments to organizational budgets due to PVC or the generation of additional external funding for programs. Policy changes refer to organization or city-wide adoption of plans and/or guidelines. Environmental changes were mainly related to additions or improvements to the built environment and surrounding infrastructure.

Individual level behavior and health outcome data were obtained from each Collaborating Organization through the quarterly and supplemental reports. Each organization collected these data differently from participants, but all measures were tracked and reported in aggregate over the course of the initiative. Individual level behavior and health outcomes have been reported elsewhere [29, 30].

### Partnerships and collective impact data collection measures

#### Annual Steering Committee survey

In collaboration with the Be Well Communities team, RTI developed and administered a Steering Committee survey annually. The annual survey of all active Steering Committee members (those who attended at least one meeting the prior fiscal year) was administered via Qualtrics in March 2018 (Year 1), January 2019 (Year 2), January 2020 (Year 3), and January 2021 (Year 4; the initiative concluded on August 31, 2020, although some organizations carried over funding for programs because of COVID-related delays). The final Year 4 survey occurred 6 months after the initiative ended. The survey largely contained the same items across years and assessed the following: program activities; partnerships; the initiative’s goals, focus, and progress; Steering Committee roles; participation in the initiative; expected outcomes; sustainability; and the influence of other health-related initiatives.

The annual Steering Committee survey also assessed the 5 domains of Collective Impact. The 5 domains include common progress measures, common agenda, backbone organization, mutually reinforcing activities, and communication [17]. While the questions in some domains varied across the years, roughly 14 questions per year measured the collective impact of the initiative. Each question asked participants to rate their agreement with the given statement on a five-point Likert scale from [1] Does not describe this initiative at all to [5] Fully describes this initiative. We created a scale measure for each of the five domains of collective impact by taking the average of the participant’s ratings of the questions within that domain. The maximum score in each domain was 5; the overall score was the sum of the average ratings of each domain for a maximum score of 25 [18].

#### Collaborating organization interviews

RTI conducted interviews with representatives from Collaborating Organizations during April 2020. Nine individuals representing 4 of the 7 Collaborating Organizations with high levels of involvement in the initiative were invited to participate in 1-h, semi-structured telephone interviews to provide additional context to the annual Steering Committee survey. All representatives that were invited agreed to participate. Some organizations were not interviewed because the key personnel that were engaged in PVC had left the organization at the time of the interviews. Interviews were recorded and transcribed, and themes were identified to explain trends and contextualize quantitative findings using rapid turn-around qualitative analysis [31].

## Results

### Reach

Overall, 47% of the community was reached through 19 healthy eating and active living EBIs in the PVC CAP. The estimated number of participants each Collaborating Organization reached through their EBIs is shown in Table 3. These reach numbers are estimates because in many cases adults and youth had exposure to multiple community programs over the course of PVC, making it difficult to track precisely how many unique individuals the initiative reached.

**Table 3:** Pasadena vibrant community’s overall program reach for each collaborating organization, Pasadena, Texas, 2017 − 2021

Collaborating Organization	Participants	Overall Program Reach		
**Brighter Bites**	Adults and Youth	5,301^*^ students enrolled 21,204^*^ family members reached		
**Harris County Public Health**	Youth	1,333^*^ students reached by the OLE! Texas program^a^ 42 staff members received Growing Up WILD and Gardening 101 training as part of the OLE! Texas program 67 students engaged in crosswalk design contest		
	Adults and Youth	3,352 students participated in Safe Routes to School walk and roll to school days 125 physical education teachers trained in Safe Routes to School basics		
**MD Anderson Center for Energy Balance**	Adults	664 participants in the Vibrant Lives weight loss program		
**Memorial Hermann Community Benefit Corporation**	Adults	292 unique clients were assisted by the community health worker 70 people enrolled in diabetes classes and 49 completed diabetes education classes 123 people enrolled and completed other health education workshops 3 additional community health workers from North Pasadena Community Outreach Center were trained and certified		
**Pasadena Independent School District**	Youth	1,384 elementary and middle school students reached by ACE CATCH Kids Club^b^ programming 87 teachers at 19 middle, intermediate, and high schools received physical education equipment and training 251 unique teachers and staff were trained on movement-based learning strategies 54,000 elementary, middle, intermediate, and high school students were impacted by programming each year for three years		
	Adults	12,591^*^ Pasadena Independent School District employees participated in a worksite wellness program		
**City of Pasadena Parks and Recreation Department**	Adults	285 unique participants enrolled in the classes		
	Adults and Youth	150 residents and 300 children reached through new playgrounds		
**YMCA of Greater Houston**	Youth	446 students at 4 campuses participated in YMCA extracurricular soccer programming 7 participants enrolled in Healthy Weight and Your Child^c^ and 4 completed the program		
	Adults and Youth	194 participants enrolled in 19 walking clubs		
	**Year 1**	**Year 2**	**Year 3**	**Year 4**
**TOTALS**	50,248^a^ individuals reached 385 teachers or staff members trained	99,135^a^ individuals reached 385 teachers or staff members trained	71,759^a^ individuals reached 331 teachers or staff members trained	54,158^a^ individuals reached

^*^Indicates participant counts are not mutually exclusive

^a^Texas Children in Nature Network. Outdoor Learning Environment (OLE! Texas)

^b^CATCH.org. CATCH Kids Club After School Curriculum Grades K-5. 2021

^c^YMCA of the USA. Healthy Weight and Your Child. 2022

The total number of individuals reached increased over the first 2 years of the initiative and dropped slightly for Year 3, which was likely caused by the COVID-19 pandemic and associated lockdowns and social distancing mandates, which restricted 8 planned program activities. Despite these restrictions, PVC continued implementing systems changes and expanded programs to additional venues—such as school campuses and parks, as permissible while doing so in an acceptably safe manner.

In response to interviews with program staff and open-ended questions on the annual Steering Committee survey, participants perceived PVC’s reach as extending beyond the specific target audiences and into the larger community.

Given the differences in measures available to evaluate how the initiative affected individual level behavior outcomes and changes in healthy eating and active living among program participants, those results are documented elsewhere [29, 30].

### Systems-level changes

PVC’s efforts resulted in professional practice, funding, policy, and environmental changes. Three of 7 Collaborating Organizations described changes in professional practices related to training staff to promote healthy food consumption, physical activity, and healthy weight. Representatives from 3 of the 4 Collaborating Organizations interviewed described professional practice changes related to organizational processes and procedures. For example, new recess guidelines in PISD were issued based on garnering buy-in from staff and parents. Additionally, PARD developed a new registration system whereby individuals could register for classes at any recreation location, which previously could only be done at one location.

Many of the funding changes reported in the interviews were efforts to seek additional funding rather than actual changes in funding. For example, PARD received external funding for shade structures and utilized volunteer time to identify grant opportunities and mechanisms. PISD leveraged PVC funding to receive a match from an organization to build playgrounds and committed internal resources to support wellness programming for employees district wide.

Three official local policy changes occurred over the 4 years of PVC and all supported PA. These included PISD and the City of Pasadena Planning Commission passing a Pasadena Safe Routes to School Plan, the City of Pasadena City Council adopting a Pasadena Healthy Parks Plan to improve parks for the first time in over 20 years, and PISD implementing district-wide recess guidelines on all elementary campuses.

All 4 Collaborating Organizations participating in the interviews noted changes to the built environment as a significant factor in long-term sustainability. These changes included the creation of 2 additional walking tracks, 7 crosswalks, 4 playgrounds, garden beds constructed at 10 school campuses, as well as park and sidewalk improvements. All organizations perceived these changes as beneficial for the community. Harris County Public Health noted that these environmental improvements contribute to increasing PA as well as to social and emotional benefits of being in nature (e.g., reducing stress and anxiety, increasing community cohesion). Similarly, PISD reported that the playground improvements at their campuses increased PA and promoted social emotional learning. Although the evaluation was not able to assess the broader community impacts directly, these comments suggest the impact of the environmental changes and are consistent with a multicomponent obesity prevention intervention that includes safe and attractive places for PA.

### Partnerships and collective impact

#### Partnerships

Throughout the 4 years of PVC, Steering Committee members completing the annual Steering Committee survey reported a strong positive impact of the initiative on new and existing partnerships. Most participants surveyed somewhat or strongly agreed in Years 1 to 4 that through PVC they: developed new partnerships in the community (92.9%), found new ways to leverage existing partnerships in the community (92.0%), connected with individuals who have positively impacted (or will positively impact) their organization’s work (98.3%), delivered programs more efficiently because of the partnerships formed or enhanced as part of this initiative (100%).

Overall, Collaborating Organizations interviewed viewed the value of PVC’s collaboration as connection, communication, and cooperation that allowed partners to address challenges and share resources to deliver their programs. Through the initiative, organizations recognized how they could help each other and rely on each other’s resources; for example, one interviewee stated:

*I think the biggest benefit is that we have found that we can help each other in ways that we may not have necessarily thought …. You talk to [partners] regularly, you have your little sidebars after the meetings. It strengthens those relationships and again, it allows you to know what’s going on with everybody else and what resources they may have that you did not necessarily know they had.*

Four Collaborating Organizations interviewed (100%) reported strengthening existing partnerships by developing relationships and increasing communication between programs participating in the initiative. Four additional organizations reported developing new formal and informal partnerships through the initiative, on the stakeholder survey. These partnerships allowed for increased pounds of food distributed in collaboration with the Houston Food Bank, enrollment in soccer clinics, delivery of diabetes workshops at additional locations, and referrals to the community health worker program.

Two organizations indicated their partnerships worked well because they shared a common agenda, which was defined by the CAP. One interviewee noted that “I think we’re all trying to accomplish the same goal. That creates just that sense of trust that we know that we have each other’s back.” Organizations described the importance of the Be Well Communities team’s role in fostering partnerships by connecting organizations and setting an expectation for collaboration.

#### Collective impact

**Fig. 2:**
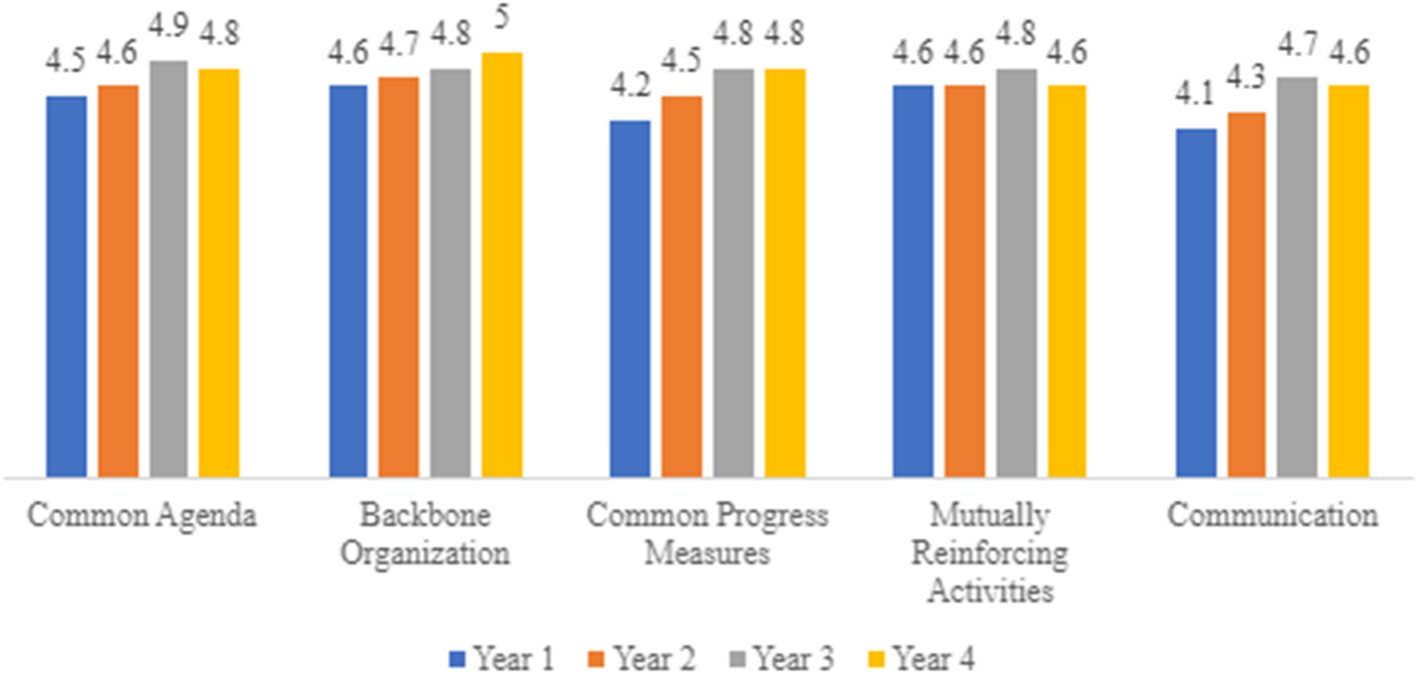
Collective impact results by domain, year 1 to 4

The 5 domains of Collective Impact—common progress measures, common agenda, backbone organization, mutually reinforcing activities, and communication—were successfully implemented and met [17], as assessed in the annual Steering Committee survey. Each domain received an average score of 4.5 or greater across all time points (on a scale from 1 to 5)—see Fig. 2. Average scores on the assessment examining the 5 domains of Collective Impact included in the annual Steering Committee survey were high, increasing from 22.0 (out of a maximum score of 25) in the first year of the initiative to 24.0 in its final year. In Year 4, all participants indicated that each of the 14 items that assessed Collective Impact mostly or fully described this initiative.

During the interviews, members of Collaborating Organizations described examples of their experiences with each Collective Impact domain; for example:

*… the overall objectives and goals that [MD Anderson] set for the partners of this collaborative, to identify clear-cut goals and expectations for all the partners I think was instrumental in being able to set the standard for all the partners in the collaborative to come together…*

Collaborating Organizations had positive views regarding PVC’s Collective Impact. They reported that the initiative fostered multisector collaborations and strong coordination with the Healthy Living Matters initiative led by Harris County Public Health.

PVC laid the groundwork for ongoing collaboration and was successful in transitioning a new organization, the City of Pasadena PARD, to take on the role of serving as the backbone organization going forward, a key component of the initiative’s sustainability. The results of the final Steering Committee survey indicated confidence that community collaborations would be sustained because Collaborating Organizations had a common goal and shared resources. By continuing to facilitate these partnerships through the Partnership for a Healthy Pasadena, the new initiative led by PARD, PVC can be expanded and sustained.

## Discussion

PVC sought to engage organizations in Pasadena, TX, to implement community-based strategies to promote healthy diet and PA, and ultimately to reduce risk for chronic disease. The evaluation findings demonstrated that the 4-year initiative was successful in initiating a dynamic dialogue about the importance of health behaviors and creating and advancing community-based strategies to inform policies that promote health. Organizations successfully implemented EBIs and made progress toward important health outcomes despite major challenges, including weather emergencies and the COVID-19 pandemic.

Population-wide reductions in risk for chronic disease require a comprehensive approach in which behavioral changes are promoted across multiple levels of the social ecological model, in multiple venues, and supported by a robust network focused on facilitating these changes [8, 32, 33]. The environmental interventions implemented as part of PVC resulted in construction of community tracks and playgrounds, school gardens, and more.

Qualitative findings from PVC interviewees indicated that including environmental and policy approaches further bolstered individual level community-based health interventions [22]. The extant literature has shown that policy and environmental changes can reduce barriers to PA and healthy diet [34–36]. The Healthy Communities Study, for example, demonstrated that community activities, programs, and policies were linked to improved childhood diet and nutrition [37]. Others have shown that EBIs have the potential to reinforce one another and yield greater and more sustainable effects than programs that target only one level of influence [8, 32, 38].

The Be Well Communities team tailored the initiative to Pasadena by including working with the Steering Committee and Collaborating Organizations, which formed a multi-sector coalition for health in the community. This approach has been utilized in other contexts; Shape Up Somerville [39] included specific strategies for healthy eating and active living coupled with a community-driven collaborative coalition with enhanced capacity to deliver programming. Delivery of EBIs must be placed effectively in the context of individuals’ lives, and community-driven coalitions can help adapt to those contexts [21].

Community-wide, multisectoral health efforts benefit from having a backbone organization providing expertise and capacity building. Serving as the backbone organization for PVC, the Be Well Communities team provided support to Collaborating Organizations to help them build the capacity needed to create changes in organization systems and the broader community. The importance of having a strong backbone organization is highlighted by the qualitative findings indicating that the Be Well Communities team served as the core organizer, catalyst, and enabler of PVC. Be Well Communities has demonstrated previous success as a backbone organization for other community-wide health initiatives. Detailed elsewhere, the inaugural community that implemented Be Well Communities showed, among other outcomes, increased access to healthy food, increased healthy food consumption, and increased physical activity in certain groups [18].

Limitations of the programs and accompanying evaluation include the challenge to realize population-level changes in chronic disease indicators over a short timeframe and balancing the collection of health outcome data with the practicalities of community-based programming. Related, each Collaborating Organization utilized different metrics to measure health outcomes in their respective populations, thus data could not by synthesized across programs. Additionally, the final year of the implementation and evaluation were disrupted because of the COVID-19 pandemic.

## Conclusion

Understanding PVC and its impacts offers an example for the development of other community-based initiatives. Implementing community-wide, multiyear, coordinated initiatives is ambitious and program implementers need to take advantage of the enthusiasm of collaborating organizations while managing expectations for progress. It is important to support organizations to help them develop realistic objectives, to understand the impact they can expect within specified time periods after implementation, and to think through how to measure impact. The PVC CAP served as the common agenda for the initiative, galvanizing a strong network of organizations who continue to lead work promoting wellness throughout the community.

## Data Availability

The datasets used and/or analyzed during the current study are available from the corresponding authors on reasonable request.

## Abbreviations

CAP: Community action plan
PARD: City of Pasadena Parks and recreation department city of Pasadena
PISD: Pasadena independent school district
PVC: Pasadena vibrant community
PA: Physical activity
MVPA: Moderate to vigorous physical activity
